# A qualitative evaluation of the national rollout of a diabetes prevention programme in England

**DOI:** 10.1186/s12913-023-10002-y

**Published:** 2023-09-29

**Authors:** Lisa Brunton, Claudia Soiland-Reyes, Paul Wilson

**Affiliations:** 1 Division of Population Health, Health Services, Research and Primary Care, School of Health Sciences, The University of Manchester, Williamson Building, Oxford Road, Manchester, M13 9PL UK; 2 Medical Directorate, North West Ambulance Service NHS Trust, Ladybridge Hall, 399 Chorley New Rd, Bolton, BL1 5DD UK

**Keywords:** Diabetes, Prevention, Implementation, England, COVID-19, Qualitative Evaluation

## Abstract

**Background:**

The National Health Service Diabetes Prevention Programme (NHS DPP) was commissioned by NHS England in 2016 and rolled out in three ‘waves’ across the whole of England. It aims to help people with raised blood glucose levels reduce their risk of developing type 2 diabetes through behaviour change techniques (e.g., weight loss, dietary changes and exercise). An independent, longitudinal, mixed methods evaluation of the NHS DPP was undertaken. We report the findings from the implementation work package: a qualitative interview study with designated local leads, responsible for the local commissioning and implementation of the programme. The aim of the study was to explore how local implementation processes were enacted and adapted over time.

**Methods:**

We conducted a telephone interview study across two time-points. Twenty-four semi-structured interviews with local leads across 19 sampled case sites were undertaken between October 2019 and January 2020 and 13 interviews with local leads across 13 sampled case sites were conducted between July 2020 and August 2020. Interviews aimed to reflect on the experience of implementation and explore how things changed over time.

**Results:**

We identified four overarching themes to show how implementation was locally enacted and adapted across the sampled case sites: 1. Adapting to provider change; 2. Identification and referral; 3. Enhancing uptake in underserved populations; and 4. Digital and remote service options.

**Conclusion:**

This paper reports how designated local leads, responsible for local implementation of the NHS DPP, adapted implementation efforts over the course of a changing national diabetes prevention programme, including how local leads adapted implementation during the COVID-19 pandemic. This paper highlights three main factors that influence implementation: the importance of facilitation, the ability (or not) to tailor interventions to local needs and the role of context in implementation.

**Supplementary Information:**

The online version contains supplementary material available at 10.1186/s12913-023-10002-y.

## Introduction

Approximately 3.9 million people are diagnosed with diabetes in the UK, of which 90 percent are diagnosed with type 2 diabetes [1] and a further 5 million people are estimated to have non-diabetic hyperglycaemia (raised blood glucose levels) in England [2]. Type 2 diabetes is a major public health concern and people with type 2 diabetes are at risk of developing complications [3]. However, type 2 diabetes is a largely preventable disease through maintaining a healthy weight, eating a healthy diet and exercising [4]. A worldwide focus on prevention of long-term conditions has gathered pace and, in the UK, the National Health Service (NHS) Five Year Forward View (2014) [5] set out an ambitious plan to undertake a national roll out of ‘Healthier You: the National Health Service Diabetes Prevention programme’ (NHS DPP).

### The NHS DPP intervention

The NHS DPP is an evidence based lifestyle behaviour change programme, and targets adults aged 18 years or older who are considered to be at risk of developing type 2 diabetes and diagnosed with non-diabetic hyperglycaemia (raised glycaemia between 42–47 mmol/mol). The NHS DPP aims to reduce an individual’s risk of developing type 2 diabetes through weight loss, dietary change and increase in physical activity [6].

The programme is delivered according to a national service specification [6, 7]. The main intervention is a group based face-to-face service delivering a minimum of 13 group-based sessions over a minimum of 9 months. The programme is delivered in various community venues by one of the national providers (see NHS DPP commissioning arrangements section below). The paper by Hawkes et. al. [8] provides findings from an observational study comparing service delivery of the NHS DPP with observed patient experiences. From 2019, an online digital service was also offered, as an alternative to group-based face-to-face sessions [6].

Referral into the NHS DPP occurs via general practice; people with non-diabetic hyperglycaemia are identified through general practice register searches or through the NHS Health Checks and offered the programme via letter, phone call or during a consultation. During the COVID-19 pandemic, a self-referral route into the programme was also initiated.

### NHS DPP commissioning arrangements

Delivery of the NHS DPP is procured through a national competitive process, organised by NHS England (NHSE). The NHS DPP is commissioned across geographically defined local sites, covering the whole of England. National providers are commissioned by NHSE, with each local site choosing one of these providers through a mini competition process to select the most suitable provider for their local needs. Each site has a site lead, usually a commissioner, who is responsible for local implementation of the NHS DPP although NHSE directly holds the contract with their provider. Site leads support general practices to identify and refer patients into the programme, and work with their chosen provider to meet NHSE expectations for uptake to the programme. Sites were given additional financial resource in the region of £30,000-£60,000 to support implementation throughout the early years of the NHS DPP roll out [9].

### Timeline of the NHS DPP roll out

The NHS DPP was rolled out across England in three ‘waves’. From May 2016, 27 areas covering a population size of 26 million people, implemented the NHS DPP (wave 1) [10]. A further 13 areas implemented the NHS DPP from April 2017 and this covered a further 25 percent of the population of England (wave 2) [11]. From April 2018, the NHS DPP roll out was extended to all remaining areas in England that were not currently included in the programme (wave 3) [11]. Four providers were chosen by NHSE to provide the behaviour change programme across England [12] and a framework contract was published in August 2016, setting out the national service specification for providers [7]. During this time, a digital DPP pilot was rolled out across eight pilot sites [13]. In 2019, the NHS Long Term Plan committed to doubling the funding of the NHS DPP over the next five years; this included increasing capacity of the NHS DPP from 100,000 to 200,000 places per year and to include a digital option to widen choice for patients and target inequality [14].

From August 2019, a new contractual framework was published, setting out a new national service specification [6]. Five providers were chosen to deliver the face-to-face group service by NHSE to provide the NHS DPP across England: four of the previous providers plus one new provider. Four out of the five providers are commercial entities and one is a social enterprise organisation. An analysis on participants to the NHS DPP from June 2015 to December 2018 suggested that, compared to White ethnic groups, participants from some ethnic minorities were 25% less likely to complete the programme, had smaller reductions in their HbA1c and lost less weight [12]. One of the aims of the 2019 framework was to improve uptake and adherence, including better targeting of populations (such as minority ethnic groups, deprived populations and working age population) to ensure equity of access onto the programme. It also addressed delays associated with running courses in rural areas. The most notable change from the previous contractual framework was the inclusion of an online digital service offer as an adjunct to face-to-face group delivery. At the time that the 2019 contractual framework was commissioned, results from the Digital DPP pilot were still emerging, and a cap of 20 percent was set to the number of digital referrals offered; before being offered the online digital programme, individuals had to be offered, and decline, face-to-face group sessions. The new contractual framework was operational from August 2019 in nearly half of the local sites (wave 4).

From 20^th^ March 2020, due to the impending lockdown in England caused by the COVID-19 pandemic, all face-to-face group sessions stopped and participants were given the option to join remote group sessions (video conferencing and telephone consultations), or pause their programme until face-to-face sessions could resume [15]. New participants and those who had yet to start their intervention were offered a choice of remote group sessions or the online digital programme (including in areas that had not yet moved onto the new framework) and the 20 percent cap on digital service allocation was lifted [15]. In response to the reduced availability of blood tests during the COVID pandemic, eligibility into the programme was changed to include any individual with an eligible glycaemic test result within 24 months instead of 12 months. The wave 3 areas that were due to move on to the 2019 contractual framework from May/June 2020 had their current contracts extended until the end of 2020.

**Fig. 1:**
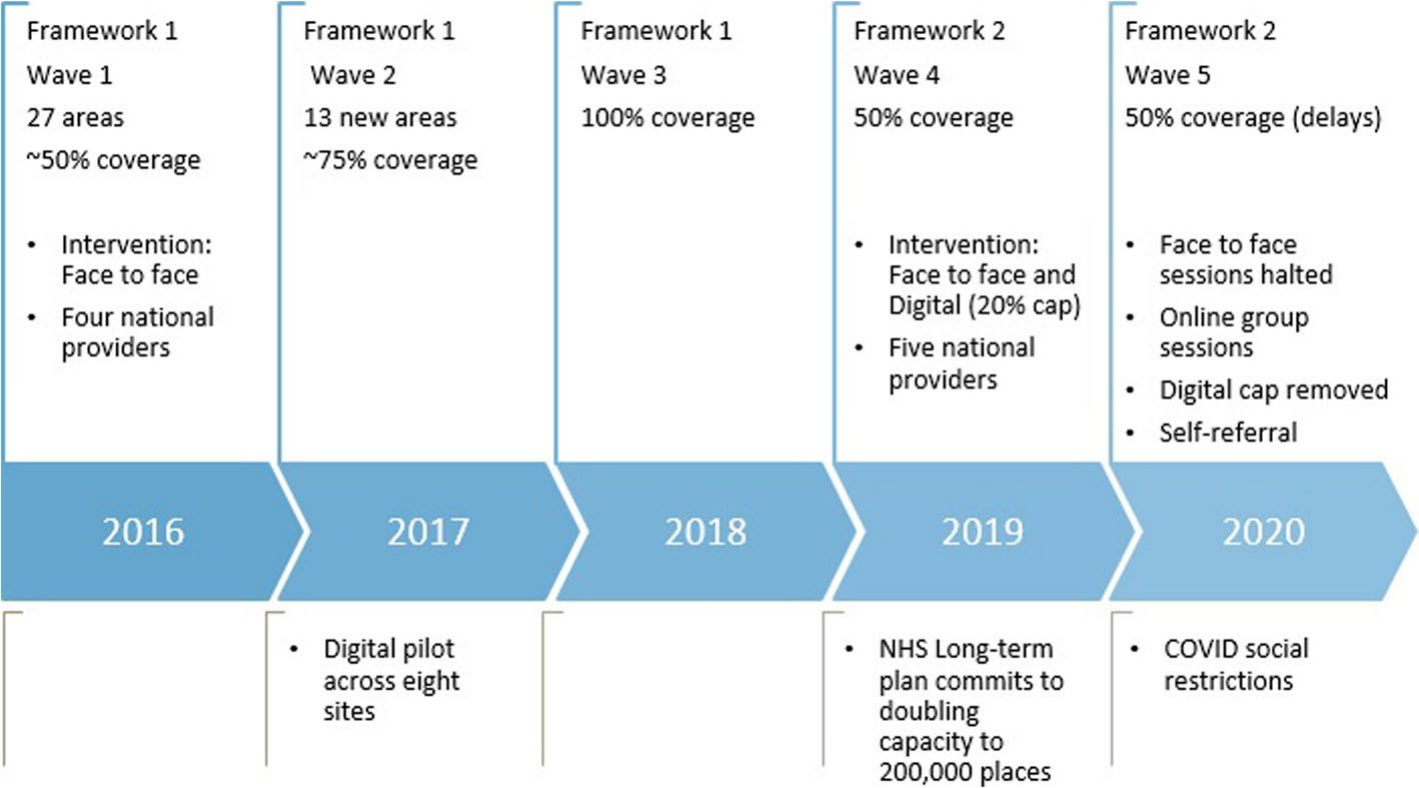
Timeline of the NHS DPP

From July 2020, in addition to the usual general practice referral processes, NHSE introduced a national ‘direct to consumer’ or ‘self-referral’ route into NHS DPP, whereby individuals were able to assess their risk of type 2 diabetes via the ‘Know Your Risk’ tool on the Diabetes UK website and, if eligible, were able to refer directly onto the NHS DPP [16]. Figure 1 outlines the contractual roll out of the key service elements of the NHS DPP.

We have undertaken an independent, longitudinal mixed methods evaluation of the national roll out of the NHS DPP to evaluate the implementation and population impact of the programme: Diabetes Prevention – Long term Multimethod Assessment (DIPLOMA) [17]. DIPLOMA suggests that the NHS DPP has reduced rates of population level incidence of type 2 diabetes [18].

A previous paper published from the DIPLOMA study presented findings from interviews conducted with local leads early on in the implementation process [19]. The authors identified lessons for future implementers to improve uptake and sustainability of such programmes; these included the need to set out clear responsibilities for all stakeholders ahead of implementation, ensure that local leads engaged early with new providers, enable a mechanism to share learning across sites, to provide guidance on the use of incentive payments to increase referrals, and raise awareness of the programme among the public and professionals [19].

This paper presents findings from the implementation work package of the DIPLOMA study [17]. It reports findings from interviews conducted at two time-points (following implementation of the new contractual framework in 2019) with designated leads responsible for the local commissioning and implementation of the NHS DPP. The aim of the study was to explore how local implementation processes were enacted and adapted over time.

## Methods

### Design

We conducted a semi-structured telephone interview study at two time-points, with designated leads responsible for the local commissioning and implementation of the NHS DPP, to explore the process of local implementation and to understand how implementation plans change over time. We wanted to recruit respondents from across England and so chose to conduct telephone interviews as an efficient means of data collection and to help ensure that any potential research burden on service delivery was minimised. Respondents chose a time that was convenient for themselves when they were able to speak privately and without interruption. We encountered no challenges with sound quality when undertaking telephone interviews.

We used an interview topic guide during interviews, although interviews were also guided by participants’ discussion during interview (for information on sample questions, see supplementary file 1). We report the interview study according to SRQR guidelines [20] (see supplementary file 2). Ethical approval was obtained from North West- Greater Manchester East Research Ethics 424 Committee (ref: 17/NW/0426).

### Participants

A sampling strategy was devised to provide adequate case sites to longitudinally evaluate implementation of the NHS DPP. Prospectus documents, which were prepared by sites for providers to review ahead of bidding for contracts, were obtained from NHSE. These documents were reviewed to help generate a purposive sample of 22 case sites. We aimed to recruit a mix of case sites which varied in terms of characteristics such as the NHS DPP provider chosen, rural and urban locations, populations with different socio-economic characteristics, local recruitment and incentive strategies, and to include sites that had moved onto the new contractual framework in August 2019.

We obtained contact details for the designated leads at each sampled case site from the NHS DPP national management team at NHSE. We sent email invites (including participant information sheet) to the identified designated leads; where more than one lead was identified, invites were sent to all named leads so that all those involved in local implementation had the opportunity to take part. Reminder invite emails were sent to non-responders after three weeks. Potential respondents replied to interview invites to indicate their interest in taking part, usually via email, and subsequently, a day/time was arranged to conduct the interview. We sent a consent form (electronically or through the post) to each respondent to complete before the telephone interview took place.

### Data collection

We conducted 24 time-point 1 interviews with 26 designated local leads across 19 sampled case sites; in six case sites, more than one local lead agreed to take part. Interviews were conducted between October 2019 and January 2020 and lasted between 24 and 75 min. Interviews were timed so that data collection started three months after the new contractual framework in August 2019 was operationalised, to explore how implementation processes had changed. Interviews were conducted by LB, an experienced qualitative research associate. At the end of the interview, LB gained permission from respondents to contact them at a later date, to request a follow-up interview.

At time-point 2, LB conducted 13 interviews with 14 designated local leads across 13 case sites; in one case site, two respondents (one new in their role) took part in the same interview, as respondents were transitioning roles. In two other case sites, new designated leads were interviewed having recently taken over the role from previous respondents. However, most designated leads at time of interview had more than two years’ experience of being the local lead. We delayed time-point 2 data collection from April 2020 due to the COVID-19 pandemic. Respondents from two case sites did not respond to follow up interview requests and four replied that they were too busy with COVID-19 pandemic response plans to take part. Interviews were conducted between July 2020 and August 2020 and lasted between 21 and 72 min. Table 1 outlines the (number of) interviews conducted across case sites.

**Table 1:** Interviews conducted across case sites

Case site number	Moved to new framework in August 2019?	Time-point 1 interview conducted?	Time-point 2 interview conducted?
4	√	√	˟
5	√	√	√
7	√	√	√
8	˟	√ 2 interviews conducted	√
9	√	√ 2 interviews conducted	√
13	˟	√	˟
15	˟	√	√
17	√	√ 2 interviews conducted	˟
25	√	√	√
29	√	√ 1 interview conducted, 2 respondents	˟
39	√	√	√*
43	√	√	√
44	√	√	√
45	√	√	˟
46	√	√ 2 interviews conducted, 3 respondents	√
47	˟	√	√ 1 interview conducted, 2 respondents
48	√	√	˟
49	√	√ 2 interviews conducted	√
50	√	√	√*

√*new designated local lead interviewed

Interviews at both time-points aimed to explore designated leads’ implementation experience and, where relevant, explore the differences between the two contractual framework processes to investigate how local organisation of the programme had changed (if at all) and with what consequences (anticipated or unintended).

### Data analysis

Interviews were digitally audio-recorded (with permission) and transcribed by a University approved transcription service. Interview transcripts were checked for accuracy and anonymised by LB before being uploaded to the Nvivo 11 software programme to aid with analysis. We undertook a thematic analysis approach [21]. LB initially coded the interviews, using inductive coding, which involved deriving the codes from the data, as opposed to deductively fitting the data to pre-existing codes [21]. We undertook interim analyses for time-point 1 interviews ahead of conducting time-point 2 interviews. This helped us to be alert to emerging categories/themes when undertaking second interviews, whilst keeping an open mind to emerging issues [22]. Later on in the analysis process, we used the constant comparative method [23] to further explore the data, identifying similarities and differences within and across the data to expand the boundaries of categories generated from interviews at both time-points. LB and PW held regular research team meetings to scrutinise the data and refine the categories to generate the overarching themes presented.

## Results

We have identified four themes that show how implementation was locally enacted and adapted across the sampled case sites over time: 1. Adapting to provider change; 2. Identification and referral; 3. Enhancing uptake in underserved populations; 4. Digital and remote service options. Supplementary file 3 outlines the emerging categories.

Data in the form of supporting quotes for each theme are included in supplementary file 4.

### Theme 1: Adapting to provider change

Fifteen out of the 19 sampled case sites transitioned to the new contractual framework in August 2019 (wave 4) and, following a procurement process, most sites switched to new providers. Most respondents described closer working relationships with providers with many reporting that greater transparency in provider contracts and better information flows for monitoring programme delivery had improved relations compared to the first contractual framework.

Most respondents said they experienced a ‘smooth transition’ to their new provider. A ‘smooth transition’ is attributable to the actions of designated local leads alongside the actions and support of other stakeholders: outgoing and incoming providers, Clinical Commissioning Group (CCG) leads, and NHSE regional and national teams.

The actions of designated local leads included having strong project management processes in place, such as bringing all stakeholders together during regular transition meetings before, during and after transition to the new provider. These regular meetings were perceived to have fostered an atmosphere of accountability that ensured that tasks allocated to various stakeholders happened on time.

Local leads used their prior experience of managing providers to enable a smoother launch this time round, by understanding and navigating previous challenges encountered. This meant they engaged early with colleagues whose input was needed to make the transition successful; for example, engaging with IT services to change the NHS DPP referral forms on General Practice systems, and collaborating with local CCGs’ communications teams to alert general practices of the change in provider.

Although most respondents described a straightforward transition, some sites still encountered challenges. The main challenge related to outgoing providers accruing significant numbers of patients on waiting lists prior to the end of their contract. This caused extra workload for providers as patients on waiting lists had to be contacted regarding change in provider and this had to be managed in a way that complied with information governance arrangements. Local leads described how providers wrestled with the decision of whether to take an opt-in approach (details sent to new provider if patient replies to give consent) or an opt-out approach (details passed to new provider unless patient replies to object). Some leads described a lack of guidance from NHSE on this issue and most providers took a cautious line and chose the opt-in approach. A significant number of patients were removed from the waiting list as a consequence. Only one case site chose an ‘opt-out’ approach, and only after significant discussion between stakeholders.

Reasons for waiting lists accruing were perceived to centre on lack of capacity. Lack of capacity was reportedly related to providers not offering venues in suitable locations or courses at suitable times, and/or waiting for specific numbers of patients to be referred from one locality before putting on a course. Waiting lists reportedly also increased when sites conducted mass mail outs if the provider was not able to cope with the subsequent demand from increased take up. Some acknowledged that waiting lists could be inflated due to poor quality referrals because of ‘pushing’ primary care for referrals, suggesting some waiting list patients were not keen to attend the programme.

Some respondents reported being unaware of waiting lists until it became a critical issue; this was perceived to be due to receiving poor quality data from providers and a hesitancy from ‘commercial’ providers to admit problems until late in the day.

Nine respondents compared their incoming providers more favourably to their outgoing providers. This was due to three inter-relating reasons: 1) a perceived improvement in communication leading to a better relationship with their new provider. This included better information exchange to help local leads target populations better; 2) improved provider capacity and resource, such as providers employing several people in roles where outgoing providers only employed one person and 3) incoming providers being perceived as more ‘proactive’ compared to their ‘reactive’ outgoing providers; this included reports of new providers putting plans in place to mitigate waiting list backlogs. Positive views regarding providers were sustained at time-point 2 interviews; with local leads reporting providers’ continued involvement in working to engage general practices to increase referrals.

### Theme 2: Identification and referral

At time-point 1 interviews, respondents described greater awareness of the programme in primary care over time, but most still acknowledged a variation in engagement across CCGs and general practices within their areas.

At time-point 1, case sites were focused on targeting low referring general practices to raise awareness of the programme and to offer support to stimulate referrals. In some case-sites, ‘outreach’ work was supported by providers who employed ‘engagement leads’ to conduct general practice visits. At time-point 1 interviews, several case sites reported that they were employing ‘facilitation officers’ to visit general practices to conduct ‘case finding’ searches (a search of general practice registers to identify eligible patients to send out letters of invitation in the post) and/or employed the services of a mailing system to organise mail shots for general practices. Making the referral process as easy as possible for professionals was considered important, given the competing demands in general practice. However, respondents at time-point 1 also indicated a desire to move away from conducting case-finding searches and mass mail shots towards a more ‘sustainable’ referral process. Some were keen to ensure the programme was ‘self-supporting’ and questioned how long NHSE would continue to provide resources to support local implementation. Respondents wanted to move towards routine identification where patients were offered the programme during routine consultations or health checks. Despite this, most patient identification reportedly did not occur directly during consultations. Given the significant uplift in referral targets that most case sites had agreed to when transitioning to the new contractual framework, this desire to shift processes appeared aspirational.

At time-point 2 interviews, local leads reported that the COVID-19 pandemic had severely negatively impacted on referrals into, and take up of, the NHS DPP across all 13 case-sites from March 2020. This was due to referral routes being suspended or severely reduced for a period. For example, far fewer patients were attending general practice for routine blood tests; most areas halted health checks; case finding searches were not undertaken, as primary care staff prioritised COVID-19 work and general practice facilitation officers were no longer able to visit practices to help undertake case finding searches. In addition, for a time-limited period (from March 2020 to approximately June 2020) respondents reported that most CCGs suspended all communications and engagement with primary care unless it was COVID-19 related. However, at time-point 2 interviews, due to the pandemic, designated leads reported using new, innovative ways to engage with primary care. For example, one site put on information webinars about the NHS DPP for general practice staff over lunchtimes and reported this led to a small increase in referrals. In addition, respondents reported that the impact of the COVID-19 pandemic had led them to develop a greater social media presence for NHS DPP locally and local leads described plans to undertake online or remote outreach work.

In response to the usual referral processes being suspended, NHSE implemented a ‘direct to consumer’ (or self-referral) route from July 2020. This received mixed views from respondents at time-point 2 interviews. Some perceived it to be a necessary interim measure to enable patients continued access to the programme. However, others expressed more negative views. Their main concern centred on ‘self-referrals’ not being counted in their referral target numbers. Other respondents questioned whether a ‘direct to consumer’ referral route could lead to greater inequalities, concerned that only the ‘worried well’ would access the programme via this route. Others perceived that it had the potential to undo all the hard work they had done to get referral processes embedded into primary care and could lead to general practice staff deprioritising general practice referral processes by relying on patient self-referral.

In our previous papers [18, 24] we reported how implementation monies were used to provide financial incentives to general practice to increase the number of referrals onto the programme. In this study we found that some case sites were still using a variety of financial incentives but that there was also within site variation in extent of use; some CCGs continued to offer financial incentives while others in the same local sites did not. In addition, some CCGs had incorporated referral onto the NHS DPP into their ‘universal service offer payments’ and this was reportedly paid for by CCG monies. However, several respondents described their desire to stop financial incentives. Reasons given included implementation monies from NHSE being reduced over time, while others felt that implementation monies were better spent on employing project managers to engage with general practice, to increase referrals.

### Theme 3: Enhancing uptake in underserved populations

The new contractual framework 2019 specified that providers must ensure equal access for all populations to reduce health inequalities, promote inclusion and support and target those with greatest need [6]. At time point 1, some case sites reported that they were still concentrating on targeting their general population to generate sufficient volumes to meet their referral targets or were in the process of identifying underserved populations within their areas, prior to any focused work being undertaken. A few sites reported working with the newly formed Primary Care Networks (PCN) to identify populations to target at a local level. In some case sites, this involved employing data analysts to analyse the practice population data.

At time-point 1, other case sites had already started to work closely with their providers to target specific local populations. This included planning and/or undertaking ‘outreach’ work, such as visiting mosques and attending community groups/events; working with local employers to target working age populations; and conducting general practice visits in areas of deprivation. However, by time-point 2 interviews, respondents reported that the COVID-19 pandemic had a significant impact on case sites’ ability to undertake outreach work and that many outreach work plans were stalled. Despite the halting of outreach work, some respondents perceived that the COVID-19 pandemic had raised the profile of diabetes prevention work. In one site, this had expedited the development of an integrated data system that would identify the demographics at risk across their PCNs, to deliver interventions (including the NHS DPP) in a tailored way. Respondents at time-point 1 reported working with providers to tailor the programme to specific populations. This included improving the provision of ‘out of hours’ face-to-face group courses for people of working age. In some areas, this was slow to be introduced, with several case sites reporting either a lack of, or inconsistent, provision of evening and weekend courses. This stemmed from providers waiting for enough ‘working age’ patients to take up the offer in a locality before they would commit to starting a programme.

At time-point 1, case sites reported working with their providers to tailor the programme to minority ethnic groups, but provision was mixed across case sites. Some respondents reported good area provision, for example, providers delivering the programme in languages other than English, or employing bi-lingual coaches who were able to translate the course for patients when required. Other designated leads described a lack of targeted or tailored delivery for minority ethnic groups. All designated leads reported that providers had developed course materials in different languages, but some did not feel that this was sufficient for equity of access. Moreover, some designated leads expressed concern that the ‘one size fits all’ standardised approach of face-to-face group sessions was a barrier to specific populations taking up the programme. Some perceived the NHS DPP to have a didactic, pedagogical approach and this was considered a barrier to attendance for people with low educational attainment and/or people whose first language was not English. Respondents reported making efforts to make courses more accessible, although some felt impeded by the lack of levers they had to enable this, given they were not the contract holders. Respondents reported wanting to make the programme more accessible to people with learning disabilities and those with mental illness. However, some perceived a lack of national support to locally modify the programme for these groups, as pilots were underway in other areas.

### Theme 4: Digital and remote service options

The digital service was a new addition to the new contractual framework from August 2019. Although some case-sites had been involved in small pilots of digital services, the online digital service was new to most of the respondents interviewed. All respondents reacted positively to the introduction of an online digital service in addition to the face-to-face group programme, perceiving it to widen access to cohorts of patients who either did not want to attend face-to-face group sessions, or who found it difficult to attend face-to-face group sessions, for example, younger (working) age populations and those who lived in rural areas.

Despite reporting positive views on the introduction of a digital offer, most designated leads reported having little knowledge about it in terms of the content of the digital service and how providers would monitor patient engagement with the service. The reason for lack of knowledge may be that designated local leads reported having minimal or no direct contact with their digital service providers (four out of the five providers did not provide the digital service directly but sub-contracted digital providers). However, some respondents were in the process of contacting their digital providers to gain more information.

At time-point 1, designated leads expressed two main concerns in the way that the online digital service was offered to eligible patients. First, respondents had concerns that patients had to turn down the face-to-face group programme before they could be offered the digital service and that this could lead to disengagement from some patients before they were ever offered the digital service. Some areas reported that there had been low uptake of the digital service in the first three months of the offer and linked this to criteria for offering digital. Second, designated local leads had concerns that the total allocation of digital places within their site could not exceed 20 percent of their overall allocated places. Respondents were concerned that the cap may be too low to meet the anticipated demand for digital and some also felt that digital uptake could have been a way to help meet their significant uplift in referral targets. Despite these concerns, most respondents acknowledged that the evidence base for the digital service was not as strong as the evidence base for the face-to-face courses and accepted the need for the cap until such evidence was gathered.

These initial concerns had shifted by time-point 2 interviews. As reported above, the delivery of the NHS DPP changed significantly due to the COVID-19 pandemic as all face-to- face group sessions were suspended and replaced with remote options. At time-point 2 interviews, remote delivery was viewed positively, and providers were praised for how quickly they got remote services up and running, and for the technological support provided to patients to enable them to take part in remote sessions. In addition, some respondents considered that the introduction of remote delivery, had made the programme more accessible for people with mobility problems and those from rural communities, as they were not required to travel to a course.

## Discussion

### Summary of findings and comparison with existing literature

NHSE implemented a national diabetes prevention programme in a phased approach from May 2016. Here we report interviews conducted at two time-points with a purposive sample of site leads responsible for commissioning and implementation of the NHS DPP across their local site. The interviews aimed to understand how local implementation processes were enacted and adapted over time to learn lessons for future practice. We chose a qualitative longitudinal research design because it is preoccupied by the concept of time when investigating the phenomenon under study; thereby, building the concept of ‘temporality’ into the research design enabled us to analyse the process of change as it happened over time [25, 26]. This paper extends our findings from an earlier paper published from interviews conducted with site leads earlier on in the implementation process [18].

Local leads described using their prior experience to put in place strong project management processes when faced with moving to a new provider. For most, this resulted in a ‘smooth transition’. These facilitation skills included engaging with relevant stakeholders, delegating tasks, and ensuring tasks were actioned to enable the change. In the integrated Promoting Action on Research Implementation in Health Services (iPARIHS) framework, facilitation is recognised as the active ingredient in implementation [27], whereby successful implementation relies on the facilitator(s) addressing factors that relate to the innovation, the recipients of the innovation and the context in which the innovation is implemented [27]. We add to this literature by highlighting the importance of local leads’ facilitation skills in the implementation of the NHS DPP.

Some local leads were frustrated by their perception of a ‘one size fits all’ approach of a national programme that left little room to tailor the programme to the needs of their underserved populations. This included groups such as those with limited English language skills, low educational attainment, or with mental health needs. Previous observational research in the DIPLOMA evaluation identified that there was variation in the way that the programme was delivered by providers [8] and that positive patient experience was more likely to occur when sessions included interactive and visual activities and were delivered in small group sizes. Patients were more likely to show dissatisfaction or disengage when sessions included complex information that was difficult to understand, when they were unable to get hold of session resources, and when group sizes exceeded 15 people [8]. Hawe and colleagues [28] argue that while there is a need to standardise the function of complex interventions, the form of individual components can be flexible and adapted to local needs. While there is evidence of adaptation to local needs in the NHS DPP [8], we found that local leads did not always consider this to be tailored enough to their local context. Barriers to the take up of type 2 diabetes self-management and support education programmes by people from underrepresented groups have been described previously [29]. This highlights how mainstream programmes, designed for the majority, do not always address the needs of minority groups. However, within the type 2 diabetes education programmes there was some evidence of tailoring programmes to fit with people’s cultural or specific needs, and this had led to increased acceptability and effectiveness in specific cohorts [29]. Nevertheless, as highlighted in the previous paper [19], this provides another direct example of how perceived tensions between local and national decision-making and implementation plays out in practice. In this paper, we have highlighted that site leads perceived there was insufficient room to tailor the programme to local needs, despite requirements on the providers to do so [6].

While the COVID-19 pandemic did severely impact on the implementation and delivery of the NHS DPP, local leads suggest that this led to an increased focus within localities on prevention work. There was recognition that people who had a diagnosis of diabetes were shown to be at higher risk of developing severe COVID-19 disease and at a greater risk of death [30] compared to people without a diabetes diagnosis. In addition, those from South Asian or Black ethnic origin were at increased risk of mortality from COVID-19 compared to those from white ethnicities [31]. Local leads identified how providers worked quickly to deliver the NHS DPP remotely and supported patients to use technology. This reflects similar reports of innovation in diabetes care during the pandemic, to transfer to a digital platform [32].

Table 2 outlines the lessons learned and the practice and policy implications.

**Table 2:** Lessons learned, practice and policy implications

Theme	Lesson learned/practice implication	Policy implication
Adapting to provider change	When transitioning to new providers, bring together relevant stakeholders in a timely fashion (including incoming and outgoing providers) and hold regular meetings to encourage accountability and ensure transition stays on track	Provide clear guidance on stakeholders’ roles and responsibilities to guide transition
Identification and referral	Good communication between local leads and providers enables better information exchange, necessary to manage provider capacity and target high need local populations	
Identification and referral	Simplify referral processes in primary care to improve identification and referral into the programme. Use of financial incentives may improve identification and referral into the programme	Implementation monies, to support implementation in the early years, are important and enable sites to tailor implementation efforts to their local context
Identification and referral	Concern that self-referral route (during COVID-19 pandemic) may increase inequalities	Monitor self-referrals to ensure inequalities are not widened
Enhancing take up in underserved populations	Work with PCNs to identify populations to target at a local level. Undertake outreach work in local community to raise awareness of NHS DPP	
Enhancing take up in underserved populations	Concern regarding lack of levers to tailor national programme sufficiently to local context	Tensions remain surrounding nationally held provider contracts versus locally held provider contracts
Digital and remote service options	Changes to programme during COVID-19 pandemic to enable digital service to be offered alongside face-to-face removed concern that patients disengage with the programme before being offered digital	Consider continuing to enable digital programme to be offered alongside face-to-face to increase access to programme

### Strengths and limitations

The main strength of this paper is the longitudinal nature of interviews, offering insights into how local leads adapted implementation efforts over the course of a changing national diabetes prevention programme. We had to delay time-point 2 interviews due to the COVID-19 pandemic and fewer interviews were conducted at this time-point, as some local leads stated they were too busy with COVID recovery plans to take part. Findings from these interviews may reflect different responses to sites that did not take part. Another potential limitation is that at time-point 2, the designated leads had changed in two case sites, and we were unable to interview the previous leads.

We chose to conduct telephone interviews as they are an efficient way to gain the data required, given we wanted to interview designated leads from across England. Face-to-face interviews are often considered the ‘gold standard’ for qualitative research [33]. Some suggest that the lack of visual cues when undertaking telephone interviews impedes the ability to build rapport with respondents and could reduce the richness of data [34]. However, we found that designated leads were comfortable with undertaking telephone interviews and this mode of data collection did not appear to impede the quality of data generated. This is supported by other studies that have compared face-to-face interviews with telephone interviews and found no significant differences between the two modalities [35].

The data was analysed inductively, using the thematic analysis approach [21] and the constant comparison method was used to rigorously interrogate the data [23]; nevertheless, the study may be limited by the lack of a theoretically informed determinants framework to guide analysis.

## Conclusion

This study highlights how designated local leads, responsible for implementation of the NHS DPP within their local site, adapted to the changes that occurred with the introduction of new service specifications introduced by NHSE from August 2019 and explores how the COVID-19 pandemic significantly influenced implementation and delivery of the programme. It adds to the literature that supports the importance of the facilitation role in implementation and identifies how local and national context can also affect the uptake and sustainability of such programmes.

This paper provides lessons for people undertaking implementation of a national disease prevention programme, such as identifying the importance of bringing relevant stakeholders together early to support changing to new providers, working effectively with providers to manage provider’ capacity, simplifying referral processes in primary care to improve identification and referral into NHS DPP, working with PCNs to identify high needs populations and undertaking outreach work to raise awareness of the programme in low referring general practices and the community.

### Supplementary Information

**Additional file 1.**

**Additional file 2.**

**Additional file 3.**

**Additional file 4.**

## Data Availability

The datasets generated and/or analysed during this study are in the form of anonymised interview transcripts. Transcripts are not publicly available but are held on a University of Manchester secure server in line with study ethical approval. Transcripts are available from the corresponding author on reasonable request.

## Abbreviations

CCG: Clinical Commissioning Group
DIPLOMA: Diabetes Prevention Long term Multimethod Assessment
GP: General Practitioner
HbA1c: Haemoglobin A1c
NHS DPP: National Health Service Diabetes Prevention Programme
NHSE: National Health Service England
PCN: Primary Care Network

## References

[1] Diabetes UK. Diabetes Prevalence 2019; Available from: https://www.diabetes.org.uk/professionals/position-statements-reports/statistics/diabetes-prevalence-2019 (Accessed on 21 August 2023).

[2] National Cardiovascular Intelligence Network. NHS Diabetes Prevention Programme (NDPP) Nondiabetic hyperglycaemia: Public Health England; 2015; Available from: https://assets.publishing.service.gov.uk/government/uploads/system/uploads/attachment_data/file/456149/Non_diabetic_hyperglycaemia.pdf (Accessed on 21 August 2023).

[3] Ninomiya T, Perkovic V, de Galan BE, Zoungas S, Pillai A, Jardine M (2009). Albuminuria and Kidney Function Independently Predict Cardiovascular and Renal Outcomes in Diabetes. J Am Soc Nephrol.

[4] Alberti KGMM, Zimmet P, Shaw J (2007). International Diabetes Federation: a consensus on Type 2 diabetes prevention. Diabet Med.

[5] National Health Service. NHS Five Year Forward View. NHS; 2014; Available from: https://www.england.nhs.uk/wp-content/uploads/2014/10/5yfv-web.pdf (Accessed 21 August 2023).

[6] NHS England. NHS DPP Service Specification 2019; Available from: https://www.england.nhs.uk/wp-content/uploads/2016/08/nhs-dpp-service-specification-aug-2019.pdf (Accessed 21 August 2023).

[7] NHS England. NHS DPP National Service Specification. 2016; Available from: https://www.england.nhs.uk/wp-content/uploads/2016/08/dpp-service-spec-aug16.pdf (Accessed 21 August 2023).

[8] Hawkes RE, Cameron E, Cotterill S, Bower P, French DP (2020). The NHS Diabetes Prevention Programme: an observational study of service delivery and patient experience. BMC Health Serv Res.

[9] NHS England. NHS Diabetes Prevention Programme: NHSDPP overview and FAQ; Available from: https://www.england.nhs.uk/wp-content/uploads/2016/08/dpp-faq.pdf (Accessed 21 August 2023).

[10] Penn L, Rodrigues A, Haste A, Marques MM, Budig K, Sainsbury K (2018). NHS Diabetes Prevention Programme in England: formative evaluation of the programme in early phase implementation. BMJ Open.

[11] NHS Digital. National Diabetes Prevention Programme Pilot Study for the collection of data from GP practices in England 2017; Available from: https://files.digital.nhs.uk/publication/8/l/diabetes_prevention_programme_-_pilot_study_findings.pdf (Accessed 21 August 2023).

[12] Valabhji J, Barron E, Bradley D, Bakhai C, Fagg J, O’Neill S (2019). Early Outcomes From the English National Health Service Diabetes Prevention Programme. Diabetes Care.

[13] NHS England. NHS Diabetes Prevention Programme – digital stream; Available from: https://www.england.nhs.uk/diabetes/digital-innovations-to-support-diabetes-outcomes/nhs-diabetes-prevention-programme-digital-stream/ (Accessed 21 August 2023).

[14] Barber S, Sutherland N. Debate Pack Number CDP 2019/0001, 7 January 2019 Diabetes. House of Commons Library; 2019. Available from: https://researchbriefings.files.parliament.uk/documents/CDP-2019-0001/CDP-2019-0001.pdf (Accessed 21 August 2023).

[15] NHS England. COVID-19: NHS Diabetes Prevention Programme 2020; Available from: https://www.england.nhs.uk/coronavirus/wp-content/uploads/sites/52/2020/03/Adaptations-to-the-NHS-Diabetes-Prevention-Programme_19-March.pdf (Accessed 21 August 2023).

[16] Diabetes UK. Type 2 Diabetes Know Your Risk; Available from: https://riskscore.diabetes.org.uk/start?gclid=EAIaIQobChMIk8HypPKd7gIVi7TtCh2BoA9TEAAYASAAEgKo1vD_BwE (Accessed 21 August 2023)

[17] Evaluating the NHS Diabetes Prevention Programme (NHS DPP): the DIPLOMA research programme (Diabetes Prevention Long term Multimethod Assessment); 2021. Available from: https://fundingawards.nihr.ac.uk/award/16/48/07#/. Accessed 21 Aug 2023.

[18] McManus E, Meacock R, Parkinson B, Sutton M. Population level impact of the NHS Diabetes Prevention Programme on incidence of type 2 diabetes in England: An observational study. The Lancet Regional Health - Europe. 2022;19:100420; 10.1016/j.lanepe.2022.100420 (Accessed on 21 August 2023). 10.1016/j.lanepe.2022.100420 PMC9160476 35664052

[19] Stokes J, Gellatly J, Bower P, Meacock R, Cotterill S, Sutton M (2019). Implementing a national diabetes prevention programme in England: lessons learned. BMC Health Serv Res.

[20] O'Brien BC, Harris IB, Beckman TJ, Reed DA, Cook DA (2014). Standards for reporting qualitative research: a synthesis of recommendations. Acad Med.

[21] Braun V, Clarke V (2006). Using thematic analysis in psychology. Qual Res Psychol.

[22] Calman L, Brunton L, Molassiotis A (2013). Developing longitudinal qualitative designs: lessons learned and recommendations for health services research. BMC Med Res Methodology.

[23] Boeije H (2002). A Purposeful Approach to the Constant Comparative Method in the Analysis of Qualitative Interviews. Qual Quant.

[24] McManus E, Elliott J, Meacock R, Wilson P, Gellatly J, Sutton M (2021). The effects of structure, process and outcome incentives on primary care referrals to a national prevention programme. Health Econ.

[25] Holland J. Timescapes: Living a Qualitative Longitudinal Study. FQS [Internet]. 2011 Sep. 16; 12(3). Available from: https://www.qualitative-research.net/index.php/fqs/article/view/1729 (Accessed on 21 August 2023).

[26] Grinyer A, Thomas C, Gubrium JF, Holstein JA, McKinney K, Marvasti A (2012). The value of interviewing on multiple occasions or longitudinally. The Sage handbook of interview research: the complexity of the craft.

[27] Harvey G, Kitson A (2016). PARIHS revisited: from heuristic to integrated framework for the successful implementation of knowledge into practice. Implement Sci.

[28] Hawe P, Shiell A, Riley T (2004). Complex interventions: how “out of control” can a randomised controlled trial be?. BMJ.

[29] Hadjiconstantinou M, Quinn LM, Tippins F, Schreder S, Khunti K, Davies MJ, editors. A perspective piece on Diabetes Self-Management Education and Support (DSMES) programmes for under-represented groups with T2DM in the UK. The British Journal of Diabetes. 2021; 21:3–10. 10.15277/bjd.2021.278

[30] Shenoy A, Ismaily M, Bajaj M. Diabetes and covid-19: a global health challenge. BMJ Open Diabetes Res Care. 2020;8(1). 10.1136/bmjdrc-2020-001450 PMC7222578 32345580

[31] Aldridge RW, Lewer D, Katikireddi SV, Mathur R, Pathak N, Burns R (2020). Black, Asian and Minority Ethnic groups in England are at increased risk of death from COVID-19: indirect standardisation of NHS mortality data. Wellcome Open Res.

[32] Quinn LM, Davies MJ, Hadjiconstantinou M (2020). Virtual Consultations and the Role of Technology During the COVID-19 Pandemic for People With Type 2 Diabetes: The UK Perspective. J Med Internet Res.

[33] Novick G (2008). Is there a bias against telephone interviews in qualitative research?. Res Nurs Health.

[34] Garbett R, McCormack B (2001). The experience of practice development: an exploratory telephone interview study. J Clin Nurs.

[35] Sturges JE, Hanrahan KJ (2004). Comparing Telephone and Face-to-Face Qualitative Interviewing: a Research Note. Qual Res.

