# Supplementary material for: A qualitative evaluation of the national rollout of a diabetes prevention programme in England
Source: BMC Health Serv Res. 2023 Sep 29;23:1043. doi: 10.1186/s12913-023-10002-y (PMC10543852; doi:10.1186/s12913-023-10002-y)
Supplement: Supplementary file 1 — Additional file 1. [file 12913_2023_10002_MOESM1_ESM.docx]

| **Topic** | **Prompts** |
| --- | --- |
| Role in implementing NHS DPP | If new contact, how long have they been in role and why was the change made? |
| Reflections on implementation of NHS DPP in their locality | Positive/negative aspects of implementation?  How local provider selected – challenges faced?  Working with local provider  How does NHS DPP fit with existing local service provision for diabetes prevention?  What other services are offered at CCG/STP level? |
| Local expectations for NHS DPP |  |
| Reflections on delivery of NHS DPP in their locality | Target populations reached?  Perceived benefits?  Perceived risks?  Unintended consequences?  Reflections on new Framework (if moved on to it) including digital offer |
| Reflections on implementation of NHS DPP into routine general practice | How has it impacted ways of working?  How are referral and clinical pathways being embedded?  Resources available to support general practice in referring people onto NHS DPP? Incentives/facilitation support?  Barriers/challenges experienced |
| Long term sustainability of NHS DPP | Any concerns?  Identify barriers/facilitators to sustainability?  Suggestions for future development of local NHS DPP? |
| Impact of COVID-19 pandemic | Changes to local implementation plans?  Barriers/challenges experienced |
