# Supplementary material for: A qualitative evaluation of the national rollout of a diabetes prevention programme in England
Source: BMC Health Serv Res. 2023 Sep 29;23:1043. doi: 10.1186/s12913-023-10002-y (PMC10543852; doi:10.1186/s12913-023-10002-y)
Supplement: Supplementary file 3 — Additional file 3. [file 12913_2023_10002_MOESM3_ESM.docx]

| **Theme** | **Category included** | **Sub category** | **Codes/Sub-codes used in category** |
| --- | --- | --- | --- |
| 1. Adapting to provider change | Respondents’ role in NHS DPP implementation  Transition to new contractual framework  Challenges in moving to new framework August 2019/transitioning to new provider    Challenges in implementing NHS DPP – provider issues | ‘Smooth’ transition  Outgoing providers accruing long waiting lists  Reduced referrals following transition  *Receiving less implementation monies in new contractual framework  Comparison between 2 providers | - Role in NHS DPP implementation - Importance of project management - How implementation monies are spent - Smooth Transition - Continuity of provider staff - Importance of project management - Engagement with/comms to primary care - Learning from previous experiences - Providers working together during transition process - Waiting list from outgoing provider - Information governance issues - Referral rates, uptake and retention rates - Receiving reduced implementation monies - Sustainability of NHS DPP – views - Capacity/resource - Data received from provider - Negative views - Escalating issues to NHSE - Responsive provider proactive provider - Working with provider |
| **2. Identification and referral** | Meeting referral targets | Referral Processes used  Challenges of meeting referral targets  Strategies employed  Impact of COVID-19 pandemic on implementation | - Referral processes - Evaluation of referral processes - ‘business as usual’ - Referral rates, uptake and retention rates - Monthly reporting halted due to COVID pandemic - Wave 3 sites – new contract delayed until November 2020 – COVID pandemic - Challenge of meeting referral targets - Capacity/resource in primary care - Differences across CCGs within an STP - Lack of engagement from general practice or CCGs - Managing underperforming CCGs or GP practices - Impact of COVID-19 pandemic on meeting referral targets - Changes to engagement with GP practices during COVID-19 pandemic - Engagement with CCGs/GP practices - Managing underperforming CCGs/GP practices - Managing referrals – over-performing practices/CCGs - Incentive payments to GP practices - Support provided to GP practices - How implementation monies are spent - Working with provider - Changes to engagement with GP practices during COVID-19 pandemic |
| **3. Enhancing uptake in underserved populations** | Targeting ‘high needs’ population | Understanding needs of ‘high risk’ populations  One size does not fit all  Impact of COVID-19 pandemic on implementation | - Meeting population targets - Meeting needs for different languages/cultures - Meeting needs for learning disabilities and mental health populations - Meeting targets? - Views on NHS DPP programme - Challenge – not being contract holders - How implementation monies are spent - Working with provider - Strategies employed to meet targets |
| 4. Digital and remote service options | Concerns over how digital is offered  Changes to programme during COVID-19 pandemic | Impact of COVID-19 pandemic on implementation | - Confidence in digital provider - Uptake of digital offer - Views on digital offer – referral process - COVID impact - Moving from face to face to remote groups - COVID impact – changes to how digital is offered - Positive views on provider during COVID-19 pandemic |
