# Supplementary material for: A qualitative evaluation of the national rollout of a diabetes prevention programme in England
Source: BMC Health Serv Res. 2023 Sep 29;23:1043. doi: 10.1186/s12913-023-10002-y (PMC10543852; doi:10.1186/s12913-023-10002-y)
Supplement: Supplementary file 4 — Additional file 4. [file 12913_2023_10002_MOESM4_ESM.docx]

| **Theme** | **Description** | **Illustrative quotes** |
| --- | --- | --- |
| **1: Adapting to provider change** | Greater transparency and better data flow from provider improved relations with providers | *WP2-22: And I think also, it’s fair to say, that NHS England were on a steep learning curve as well…a lot of the data management of the programme has changed quite significantly really. And we know more now, I think, about what the contract is, where the payment points are, what is expected of the providers, so better information flow, I personally think. Site 45, time-point 1.*  *WP2-39: I think it’s probably taken us a good two years from implementation to get good quality data so that we’ve been able to manage the contract effectively. Part of that is because the contract actually lies with NHS England rather than the [GGCs] so from the provider’s point of view, they’re trying to feed our beast in terms of reporting requirements we need and then reporting requirements that NHS England need. Site 15, time-point 1.* |
|  | Smooth transition to new provider brought about by putting in place strong project management processes, learning from previous transitions and stakeholders collaborating together | *WP2-42: …being the lead CCG involved, I’ve been responsible for the transition plan and pulling everything together. So we had a very robust transitional plan for both outgoing provider and incoming provider, as well as…between the CCGs. So we’ve basically had fortnightly calls, where we go through the plan. Site 44, time-point 1.*  *WP2-08: Because we'd done both the pilot with two different providers and then the first wave with a different provider again… So we knew about the transition process and what some of the pitfalls could be. We had the learning from that, we understood that we would need to work with our local council colleagues because they commission the NHS health check programme…so the health check template on GP clinical systems had an embedded link to the provider referral form, to make it as easy as possible for clinicians to be able to just click on the link and refer. So we knew that we'd have to work with them and with our IT colleagues in terms of disabling the old referral form and enabling the new one. Site 17, time-point 1.*  *WP2-25: Their working relationship was good and they would come to our steering group [meetings]. …[Outgoing Provider] were on the calls, they were really open and honest about any referrals or pre-initial assessments that they had […] And that really helped with the transition because obviously without that we wouldn’t have had that good transfer of information. But they worked really well so we were really fortunate with that. Site 9, time-point 1.* |
|  | Challenge – outgoing providers accruing large waiting lists prior to transition | *WP2-08: There was a bit of angst, I think, from the outgoing provider, because we opted for…an opt out option […] Now the outgoing provider was not happy with that. There was a lot of discussion and we involved the information governance … team here to look at what the implications of it were. And [regional] NHSE were part of the discussions around that as well…because on the NHSE guidance it didn't stipulate one or the other. Site 17, time-point 1.*  *WP2-22: The biggest challenge is… [providers]…work in the private sector and they had, what I would describe as a salesman approach…it’s not a way we’re used to working, we don’t want big smiles and bit promises and nothing delivered…we’d rather hear “we’ve got a problem with this and we’re not sure what to do with it” and we’ll help with that… So, yeah, the clash of culture, I think, was quite significant, because I personally feel that in the private sector the instinct is to conceal any problem, gloss over it, give it a spin. Site 45, time-point 1.* |
|  | Comparing incoming providers more favourably to outgoing providers | *WP2-08: We were quite excited actually about having a new provider, because we had struggled with the previous one in terms of communication and relationships and it was quite difficult at times... Site 17, time-point 1.*  *WP2-36: I can see that [new provider] are a much more proactive provider. You know, the kind of interactions are better. Site 29, time-point 1.* |
| **2: Identification and referral** | Variation in engagement across areas | *WP2-37: I think within primary care, you know, there is still more work to be done because not all the practices are referring patients at the numbers that could happen. There are some practices that aren’t so yeah there’s still work to be done. And obviously there are some GPs [general practitioners] that probably haven’t referred any patients and it could be due to they’ve heard there’s a waiting list or another reason so… Site 49, time-point 1.* |
|  | Using implementation monies to employ facilitation officers | *WP2-08: … the funding to support the programme is very minimal and what we spent it on was project management, but it was well spent…particularly because we had that big geographical area, four CCGs and three local authorities involved in it, you needed someone to be that local point of contact…So the funding that came with the NDPP was purely spent on project management and continues to be spent on project management. There wasn't any funding in there to incentivise practices. The incentivisation has come from the CCG…So any area starting up as a new wave…is definitely get a project manager. Site 17, time-point 1.*  *WP2-27: I mean if you’re working and if you can establish good relationships…I think the cornerstone of the programme is establishing good relationships with the GP practices, so they understand about the programme, they know the benefits to them and their patients, and it’s really easy to refer in, and that’s what we’ve done. Site 39, time-point 1.* |
|  | Making referral process easy important to ensure health professional engagement | *WP2-27: So we felt if they were able to just to…very, very easily refer a patient to the programme, literally by just clicking a few buttons, and generating a letter, that, in many respects, it’s easier for the practice than having to consider what to do next with this patient. So we sold it on that and the message got through and people took it upon themselves then to start referring and then our referrals were good and high. Site 39, time-point 1.* |
|  | Meeting referral targets set by NHSE | *WP2-29: I know we’re supposed to be looking at the most deprived areas and the BAME population. There isn’t a plan at the moment as such to pull out of the more affluent and less BAME populations, if you see what I mean, because actually at the moment it is probably those areas that are ensuring that the referral numbers are hitting where they need to hit. But certainly we will be working with the CCGs that have got the most deprived areas and ensuring that their referral numbers are increasing. It’s difficult because that’s the area that’s harder to get the referral numbers from, just because of patient uptake levels and patient engagement. Site 8, time-point 1.* |
|  | Effects on usual referral processes following COVID-19 pandemic | *WP2-45; We have found that, of course, following Covid, the number of routine HbA1c tests has plummeted and, actually, referrals into the service have dropped dramatically. Site 39, time-point 2.*  *WP2-43: So since January, obviously it was ticking along nicely in January, and then, as everyone knows, March happened and everything, unfortunately, took a bit of a halt. Obviously, the NHS had different priorities all of a sudden. People were told not to go outside, so people stopped going to the doctors. Even people that may have been at risk of developing diabetes, they didn’t go to the doctor’s because they didn’t want to catch COVID, obviously. So obviously the referrals coming into the programme dropped significantly. Site 43, time-point 2.*  *WP2-42: And in fact, we had very clear direction not to be contacting primary care and practices while they were responding to COVID. They didn’t want us sending separate correspondence, all correspondence had to go through one line of a bulletin and if it didn’t have anything to do with COVID directly, it wasn’t allowed to be in the bulletin. So, very much we couldn’t communicate the fact that these…and even if we could they wouldn’t have paid any sort of attention to that all anyway, to be honest with you…So, it was a difficult time and yes, digital was still available, but no referrals going through. Site 44, time-point 2.* |
|  | Finding new and innovative ways to engage with primary care | *WP2-30: And we’ve also put on some engagement sessions for… This is the beauty of it being virtual though, because we could never have done these practice [engagement] sessions without being able to use [Microsoft] Teams and everything. So we’ve just basically set a few Teams dates and said anybody come, any practice, any person come to any of the dates, and obviously we’re not restricted by travelling or getting time away from practice. So they’ve been quite popular, and then [our provider] have just done a standard presentation but basically done a Q&A at the end, which we think it’s been useful. We’ve had quite a good uptake, haven’t we, considering it happened in the August as well, July.*  *WP2-46: Yeah. And we’ve been able to run them over lunchtime as well. So yeah, that’s worked well. Site 47, time-point 2.* |
|  | Views on ‘direct to consumer’ or ‘self-referral’ referral route | *WP2-48: We have had a discussion this morning in our steering group on how it can affect our delivery of the programme, because what we understand is that numbers that go through to our service provider generated through the direct consumer route cannot be used towards our target…that seems a little bit ridiculous in that it consumes our provider in terms of their capacity, working towards something that doesn't meet our [ICS] target…So we have slightly mixed views, and I think some of that depends on how it can link to the delivery of our…I don't want to use the word target, I've used it a few times, but it's really that, overall it's very positive, but we need to see how it works towards our [ICS] model. Site 25, time-point 2.*    *WP2-40: … because we’ve put so much hard work in getting primary care to refer into this programme that you have in that self-referral route is, kind of, devalues that work a little bit. But I totally get it and totally understand why they’ve done it… and especially, I think, with the death rate through COVID and how many patients have been diabetic… and I suppose it’s also that patients are not being able to access primary care, what’s happening with them? Whereas they could [now] self-refer themselves through the “know your risk [tool]”. Site 5, time-point 2.* |
| **3: Enhancing uptake in underserved populations** | Identifying underserved populations | *WP2-40: We’re definitely going to be looking at doing more especially with the equity allocation. We’re just in the process of doing like a mapping exercise to find out where the pre-diabetes population, the BAME community population, and then where the practices are and where are the low referral practices. So, we can start almost identifying where the hotspots are that we need to do targeted work. Site 5, time-point 1.*  *WP2-44: And there’s a new product called [integrated data system] which is rather ground-breaking in that all providers have signed up to use it, so essentially takes all primary care, secondary care, tertiary care data and has it all in one dataset. So we’re developing our health inequalities strategy which includes prediabetes, we’re developing that to understand how we need to target the intervention at PCN level…and then we’re essentially going to be targeting the areas with the most at risk groups and tailoring the interventions to the needs of those groups…it’s got a massive boost because of the health inequality element of COVID, because it’s been identified as the key dataset in tackling that. So they’ve been given extra resource and I know the senior people on the team quite well so I got in really quickly and diabetes has become the test case for it. Site 50, time-point 2.* |
|  | Challenge – provision of ‘out of hours’ groups inconsistent | *WP2-31: [I] think we had one weekend group and four evening groups, and we have had to defer one of the evening groups… just to make sure that we’ve got the number that we needed… It’s definitely been a step in the right direction because it was the one big challenge that we had been having with [provider] for a long period of time, was getting them to make that commitment to do the evening groups. Site 13, time-point 1.* |
|  | Provision of groups in languages other than English | *WP2-35: And the actual courses are all delivered in English, they haven’t done ones in another language. So it is a bit clunky and not as easy access as we want it to be. So we are trying to, in different areas…we are trying to encourage them to put on courses for the future; they’re working up. Site 48, time-point 1.*  *WP2-37: At the moment we have…we’re very fortunate in that we have an educator [provider employee], he speaks multiple languages and they’re just in the languages that we want. So the way we’re taking it…because what we don’t want is to provide a course and only have four people turn up. So what we’re doing is we’re saying, it’s in English but what he is able to do so that people are not…the people there that don’t understand then he’s able to translate. Site 49, time-point 1.* |
|  | Challenge – ‘one size fits all’ approach to programme limits success in underserved populations, e.g. those with limited literacy levels | *WP2-22: I think also because our areas of deprivation, understandably, are areas of quite low educational attainment, higher concentrations of people with learning disabilities and these…it’s a perennial problem, these sorts of programmes are perfect, they’re accessible and wonderful for your averagely intelligent, middle class, mobile, reasonably affluent person….And that isn’t our entire population…And, yeah, how accessible it is to people who would find getting to these sorts of things harder and find understanding them harder, we’re not clear yet. Site 45, time-point 1.*  *WP2-45: …it’s very classroom based and for people who have limited levels of literacy and limited confidence in an academic approach to life this is probably going to be off-putting. […] I think you’d find that it’s perpetuating inequality, that those who are able to get themselves to the classes and have the time, can, and those that need but find that process harder and don’t see the benefits. […] So it needs a bit of an overhaul and a substantial rethink, and it needs to be in line with the NHS long-term plan personalised care approach, and building the programme around the needs of the person who’s going to be attending. Site 29, time-point 1.* |
| **4: Digital and remote service options** | Positive views expressed on digital offer | *WP2-42: My views on the digital is that it’s…I think it is very important to have that additional offer. I certainly don’t think that with the programme that’s so in depth, and a long programme, such as the NDPP is, when it’s face-to-face, that that will suit everyone, you know, depending on their lifestyle and their circumstances. And so I think to have that offer, is really important. I think it might be a way of reaching people that wouldn’t otherwise necessarily end up being involved in the programme. So I see it as a very…I see it as a positive. Site 44, time-point 1.* |
|  | Concerns regarding NHSE’s mandate on how digital services can be offered to patients | *WP2-28: Oh, I think the general feeling is that it’s still quite hard to get onto digital. You have to sort of refuse three times to go on face-to-face. I think, just talking to some of my colleagues, that I think they’d like to see digital being easier to access, rather than really having to turn down face-to-face. But I think that will…again, I think that will come. It’s very early days yet, in terms of NDPP and I think the research, a lot of the research is around the face-to-face, as opposed to the digital. Site 50, time-point 1.*  *WP2-23: We are being constantly told to push the face-to-face as the primary offer, which we understand it is more evidence based, but the digital offer is going to be more appealing to some people, and the fact that they have to kind of decline three reasonable offers of face-to-face in order then to get the digital, and they potentially have to wait a bit of time, it’s not great, but we are thankful that that option is there, nonetheless. Site 43, time-point 1.* |
|  | Positive views expressed for providers in moving to remote delivery of programme | *WP2-46: Some people it’s just not what they want to do, the virtual. Although [provider] have even said they’ll offer support for people to get onto Zoom, which is the platform they use. So they’ll offer them sessions to talk you through and do one to one sessions for people who are really nervous and things like that. So I think they’re trying to do quite a lot for people to get them onto Zoom, because the message is now it’s not going to be face-to-face for the foreseeable future. Site 47, time-point 2.* |
|  | Remote sessions widen access to programme | *WP2-01: Now, Covid has given us the opportunity, because it’s gone to a remote programme, to get rid of the waiting list to a certain extent… So, what that actually means is that for the provider they can have a coach from anywhere in the country, because it’s remote, running a programme here in [case site 7]. And also, it means that within [case site 7] we can have people from [different localities] and so on, on the same programme, whereas with the actual group--based programme you need a certain number to make [it] viable. Now this is fantastic for us; it’s fantastic for individuals because it opens it up to disabled people, because they don’t need to worry about transport and getting there. It opens it up to people who are deaf, so we’ve got some deaf people coming through, so we can do something with deaf people. And it opens it up to people being able to get put onto a programme really quickly…It opens up an opportunity for people to do the programme in their own language, so we’ve got Urdu speakers and Bangladeshi and so on. Site 7, time-point 2.* |
